# Do Current Asthma-Preventive Measures Appropriately Face the World Health Organization's Concerns: A Study Presentation of a New Clinical, Prospective, Multicentric Pediatric Asthma Exacerbation Cohort in Germany

**DOI:** 10.3389/fped.2020.574462

**Published:** 2020-11-25

**Authors:** Malik Aydin, Ella A. Naumova, Soeren Lutz, Almut Meyer-Bahlburg, Wolfgang H. Arnold, Florian Kreppel, Anja Ehrhardt, Jan Postberg, Stefan Wirth

**Affiliations:** ^1^Center for Child and Adolescent Medicine, Center for Clinical and Translational Research (CCTR), Helios University Hospital Wuppertal, Witten/Herdecke University, Wuppertal, Germany; ^2^Clinical Molecular Genetics and Epigenetics, Faculty of Health, Center for Biomedical Education and Research (ZBAF), Witten/Herdecke University, Witten, Germany; ^3^Department of Biological and Material Sciences in Dentistry, Faculty of Health, Witten/Herdecke University, Witten, Germany; ^4^Children's Hospital, Helios Hospital Niederberg, Teaching Hospital of the Essen University Hospital, Velbert, Germany; ^5^Department of Pediatric Rheumatology and Immunology, Children's Hospital, University Medicine Greifswald, Greifswald, Germany; ^6^Chair for Biochemistry and Molecular Medicine, Faculty of Health, Center for Biomedical Education and Research (ZBAF), Witten/Herdecke University, Witten, Germany; ^7^Faculty of Health, Center for Biomedical Education and Research (ZBAF), Institute of Virology and Microbiology, Witten/Herdecke University, Witten, Germany

**Keywords:** asthma, allergy, childhood, exacerbation, epigenome, biomarker, healtcare

## Abstract

In summer 2017, the World Health Organization published 10 facts on asthma, which is known as a major non-communicable disease of high clinical and scientific importance with currently several hundred million people—with many children among them—suffering from air passages inflammation and narrowing. Importantly, the World Health Organization sees asthma as being underdiagnosed and undertreated. Consequently, much more efforts in clinical disease management and research need to be spent on reducing the asthma-related health burden. Particularly, for young approximately 6 months aged patients presenting recurrent bronchitic respiratory symptoms, many parents anxiously ask the doctors for risk prognosis for their children's future life. Therefore, we urgently need to reevaluate if the current diagnostic and treatment measures are in concordance with our yet incomplete knowledge of pathomechanisms on exacerbation. To contribute to this increasing concern worldwide, we established a multicentric pediatric exacerbation study network, still recruiting acute exacerbated asthmatics (children >6 years) and preschool asthmatics/wheezers (children <6 years) since winter 2018 in Germany. The current study that has a currently population comprising 176 study participants aims to discover novel holistic entry points for achieving a better understanding of the poorly understood plasticity of involved molecular pathways and to define biomarkers enabling improved diagnostics and therapeutics. With this study description, we want to present the study design, population, and few ongoing experiments for novel biomarker research.

**Clinical Trial Registration**: German Clinical Trials Register (Deutsches Register für Klinische Studien, DRKS): DRKS00015738.

## Introduction

“Asthma deaths will increase in the next 10 years if urgent action is not taken” (1). At this point, doctors are usually left empty-handed because no definite management guidelines are deduced, currently except few prevention measures to answer questions from expectant parents. The particular emphasis of current research is mostly captured on biomarker research, and some effort is directed toward the development of antibody therapeutics in adult asthmatics or autoimmune disorders (2). The asthma prevalence had increased dramatically from 3.6% in 1980 to 7.5% in 1995 and from 8.7% in 2001 to 9.3% in 2010 in children below 18 years (3–6). The prevalence in childhood is sex- and age-dependent, i.e., preadolescent male children have an increased risk of asthma symptoms and hospitalization rates compared with girls (7). Despite remarkable progress evolved from basic research, still, 338,000 people die every single year (1). Although the asthma prevalence is higher in childhood, the asthma-associated mortality rate is increased in adults (8). Nowadays, the main goal of care for patients with bronchial asthma is quality of life improvement, whereby many of them are excluded from this aid *de facto*. Unfortunately, many patients worldwide do not have access to the current standard of treatment, and the implementation of diagnostic and therapeutic measures can be hampered by the educational and social levels of the patients and their custodians. In part, the highest lethality rates are found in economically developing countries, suggesting that underdeveloped medical care drives the increased numbers of asthma fatalities (9). This high number challenges the view that inadequate medical care is the predominant cause of numerous fatalities. Clearly, there must be other important factors being causative for this high death toll (8).

Except for some scientifically accepted prevention measures, e.g., breastfeeding or farming in childhood, scientists and clinicians also focus on describing causes that may potentially have an impact on the pathogenesis of allergy and asthma (10, 11).

Furthermore, we will have to identify and precisely characterize the triggers responsible for recurrent asthma attacks of individual children with bronchial asthma manifestation. Are discrete trigger factors sufficient for an exacerbation, or do multifactorial causes contribute to an asthma attack? Is each asthma attack clinically and/or molecularly similar? How can we classify risk profiles for those patients? How can we draft personalized therapy suggestions to parents of affected children to prevent future attacks? The main reasons for choosing these trigger factors are to understand the molecular interaction between the triggering substances and the individuals.

The pathomechanisms involved during allergic sensitization have been previously studied in murine models, as described in the literature. This fostered our current understanding of this allergic disease. In particular, the first description of airway eosinophilia and the T helper 2 cell and the associated cytokines were initially described in murine models (12, 13). Murine models enable scientists to functionally analyze processes and mechanisms (14). Persson and colleagues have exemplarily observed that interleukin-1β is involved in lung neutrophilia and T helper 2 inflammation in virus-induced asthma exacerbations in an interleukin (IL)-1β^−/−^ mouse model (15). The deriving complex data provide interesting hypotheses, enabling us to get more detailed insights into relevant asthma pathomechanisms in humans (16, 17). But so far, unfortunately, these results are currently far from translation into clinical reality and personalized healthcare. With respect to affected individuals, particularly for children and adolescents, an extremely difficult clinical all-day problem is balancing packages of measures for appropriate prevention IL-therapy options. Such individualized expedient measures would be extremely desirable because many severe asthma attacks can put so much strain on the lung that the recovery takes several weeks, with scars left behind being very likely. In detail, patients with a severe asthma exacerbation present a prolonged recovery period. The recovery time and the responsiveness to the treatment are variable here (18). A prolonged recovery may be associated with decreased prognosis and increased morbidity. O'Byrne and colleagues have previously shown that severe asthma exacerbation is associated with a declined lung function (19). Exactly, these open problems must urgently be considered and be taken to our researcher's and clinician's hearts.

## Aims and Scopes

**Figure 1:**
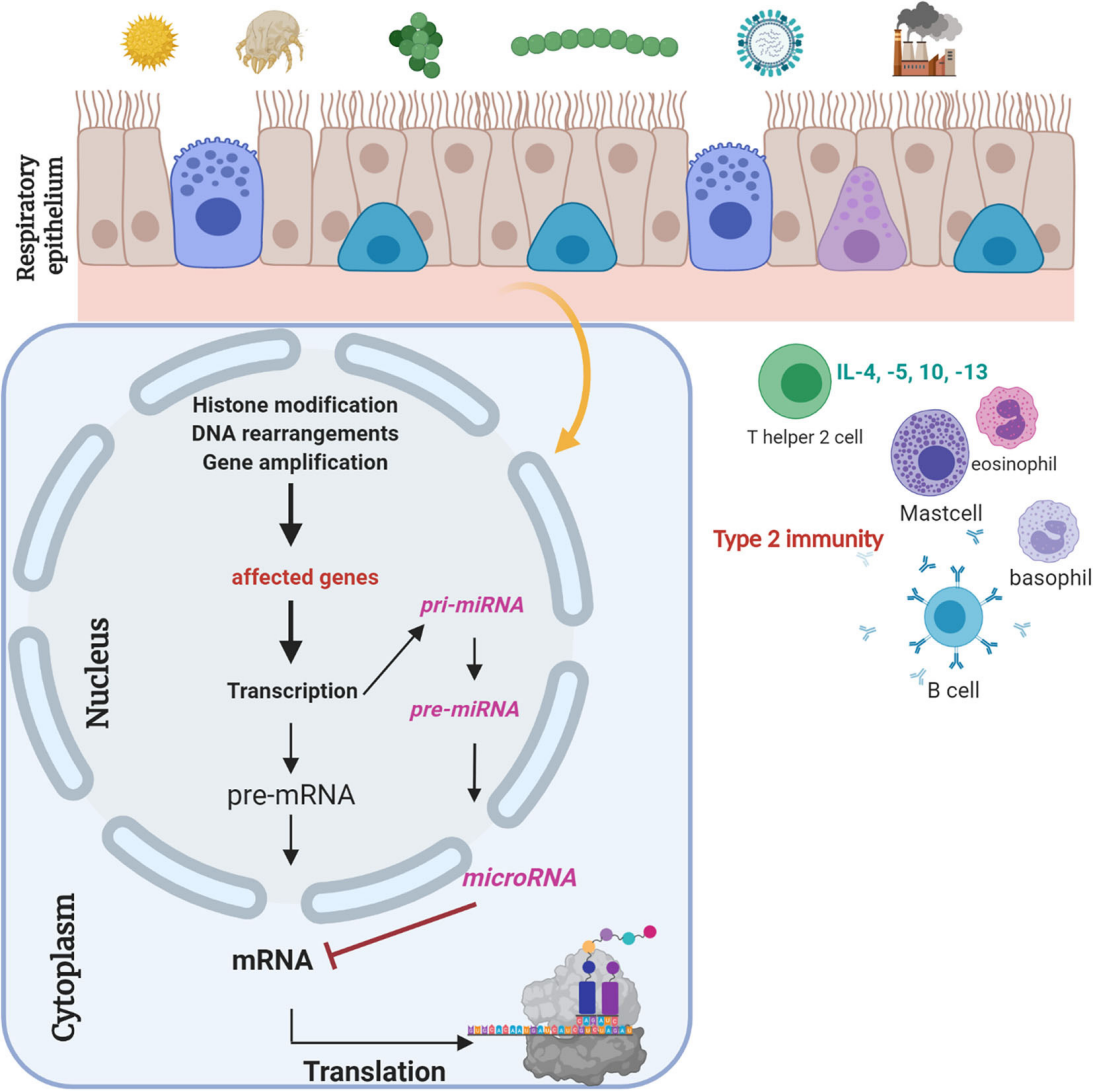
Respiratory epithelium is a gateway for “biocode” research. Respiratory epithelium is constantly connected to environmental factors. Different trigger factors can lead to epigenetic changes. Gene regulatory molecules, i.e., microRNAs, are small non-coding RNA molecules with a length of 21–23 nt, which regulate gene regulation posttranscriptionally (20). Their role and the role of siRNAs and medium and large RNA molecules as potential biomarkers will be analyzed. Experimental design of ongoing experiments to define biomarkers for clinical and therapeutic implementation. This figure was created with BioRender.com.

A major focus of our research is to gain insights into the environmentally triggered plasticity of the regulome in the nasal and bronchial epithelium—a portal of entry for many potential triggers of asthma and the epigenome of these cells as a probable molecular interface between the environment and genome interpretation. One of our main research projects is the analyses of non-coding RNA molecules (small interfering RNA, microRNA, medium, and large RNAs). To give an overview, Figure 1 summarizes the effects of extracellular trigger factors on the nasal and bronchial epithelium. The biogenesis of small interfering, medium, and large RNAs, which we also focus during our investigations, are not presented here. In detail, small non-coding RNA molecules will be holistically analyzed in the biomaterials (e.g., serum samples, nasal epithelial cells) of the cohort, and the interaction to the naso-/broncho-epithelium will be enlightened to figure their role as targets in experimental cell culture models. Our laboratory has previously established a special miR-specific isolation protocol in cooperation with the colleagues from Paris/France, which allows us to analyze all miRs in one sample through the next-generation sequencing (NGS) method. Weil et al. (2017) previously described in detail each methodological step for the microRNA characterization from biomaterials (21). In addition, we have established an adenovirus-vector system for functional analyses of the selected non-coding RNA molecules. The exposure of risk factors and the influence on exacerbation and course will be analyzed in *in vitro* models. Subjects will not undergo any interventions or allergic exposure. To implement this idea, we established an organotypic three-dimensional (3D) cell culture model of primary human nasal and bronchial epithelial cells from numerous healthy and affected study participants where these vectors can be validated. Furthermore, we will analyze these targets-of-interest in mouse models where gene therapeutic techniques, e.g., CRISPR/Cas will be used to knock-down the molecule-of-interest to present the affected signaling pathway. Here, we will also use *Dermatophagoides pteronyssinus* extracts to mimic a somewhat exacerbation model *ex vivo*. Furthermore, the effect of *D. pteronyssinus* on virus infection and on cell integrity will be tested. This project is still under publication. Here, subject-derived biomaterials are essential to perform *in vivo*-like experiments. Our major focus will be the exploitation of potential biomarkers for diagnostic use, which may possibly open new sources for therapeutic regimens in the near future.

Descriptively, observed results derived from human samples will be proven in at least cell culture models to establish their clinical and biological connection and highlight their importance for clinical routine (22–24).

Next, the microbial and fungal role of the airway epithelium in nasal and pharyngeal swabs will be explored to highlight potential preventive strategies. Through the NGS method, we will analyze the 16-S and 18-S rRNA in the nasal and pharyngeal swabs of the entire cohort to analyze the microbiome and fungiome levels of exacerbated subjects. A detailed description of the method is currently in publishing, and the details will be shared upon request. With this methodology, we will be able to observe bacteria or fungi where their cultivation with traditional culturing techniques is not possible. Furthermore, we will also start to collaborate with the asthma study network in Greece to compare here phenotype changes between the two countries.

Another focus will the exploitation of the role of IL-33 on regulatory B cells and memory B cells. Co-culture experiments will highlight this functional connection and the influence of IL-33 *in vitro*.

Then, cytokine and chemokine analyses in different biomaterials, e.g., serum, plasma, and induced sputum, will show whether an acute exacerbation is associated with specific “cytokiome” levels and whether their release can be used as potential laboratory exacerbation parameter in clinical routine. The additional biomaterials, particularly sera/plasma and cells obtained from the study cohort, may support the idea of translational research to establish biomarkers for early diagnosis of exacerbation and rapid treatment to reduce morbidity and mortality. The experimental results will be compared by correlation analyses with the clinical item parameters obtained from the questionnaires. The listed network partners (including life scientists and physicians) are clinically and experimentally experts in their area, and the methods are appropriated to implement these research questions into praxis. Most of the described projects have been done or will be performed soon.

In summary, the main focus of our clinical and experimental work is to detect new phenotypes and endotypes, evaluate developed biomarkers and to identify new biomarkers, to uncover the functions of individual cells during an exacerbation, and to decipher the characterization of the microbiome and its role during exacerbation.

Due to the significance of this extraordinary study design and population and the currently performing analyses on the biomaterials with the emphasis on different clinical and molecular levels will present comprehensive insights into the molecular “barcodes of asthma,” which might contribute to improving our understanding of relevant common and individual pathomechanisms, and as such may serve as a highly relevant example for implementation the idea “from patient to bench and from the bench back to the children's hospital” to provide a potential clinical and therapeutic translation.

## Methods and Analysis

To counteract to this concern, we have built a clinically prospective, multicentered, observational exacerbation cohort to provide deeper insights. Starting in winter 2018, we have now established a pediatric exacerbation network in Germany. The study recruitment is currently ongoing, up to date having already recruited 176 study subjects having chronic bronchitis/wheezing episodes (age group <6 years, *n* = 62), children and adolescents with bronchial asthma (age group >6 years, *n* = 55) presenting recurrent asthma attacks, as well as healthy children, adolescents, and adults (*n* = 59). Currently, our biomaterial collection comprises approximately 2,500 specimens, including sera, plasma, cells, swabs, and sputum.

The study cohort comprises two diseased groups (acute exacerbated wheezers and asthmatics) and healthy children and adults. The subjects will not undergo any interventional/allergic provocations.

The inclusion criteria for subjects with asthma are based on international recommendations, including lung function parameters (25), a gestational age over 37 weeks with an inconspicuous peripartal/postnatal period, and presenting an acute asthmatic attack at study enrollment. The definition of subjects with a wheeze is based on previously published data (26–29), including a history of at least two obstructive bronchitis episodes and acute wheezing at study enrollment, as well as an inconspicuous peripartal/postnatal period. Patients (chronic bronchitis and asthmatics) suffering from acute wheezing, respiratory illness, dyspnea, chest tightness, etc. (acute respiratory symptoms) were classified as “exacerbators.” The recruitment of wheezers is clinically and scientifically promising because underlying mechanisms of whether wheezers will develop chronic asthma are not sufficiently understood. Epigenetic signatures/environmental factors are likely to play important roles in disease development and persistence of symptoms (30, 31). We aim to analyze similarities between wheezers and asthmatics to make the diagnosis at the early stages of the disease and to discover potential biomarkers for the clinical routine. Moreover, subjects with no chronic diseases or chronic medication and an inconspicuous neonatal period were classified as healthy controls (age range: 3 months to 65 years of age). The healthy pediatric controls will be age- and sex-matched with diseased children (preschool asthmatics/wheezers and asthmatics). The recruitment of healthy adults will be helpful for correlation analyses of age-dependencies. In the future, we are also planning to include adult asthmatics. This is currently in consideration with the entire study members.

**Figure 2:**
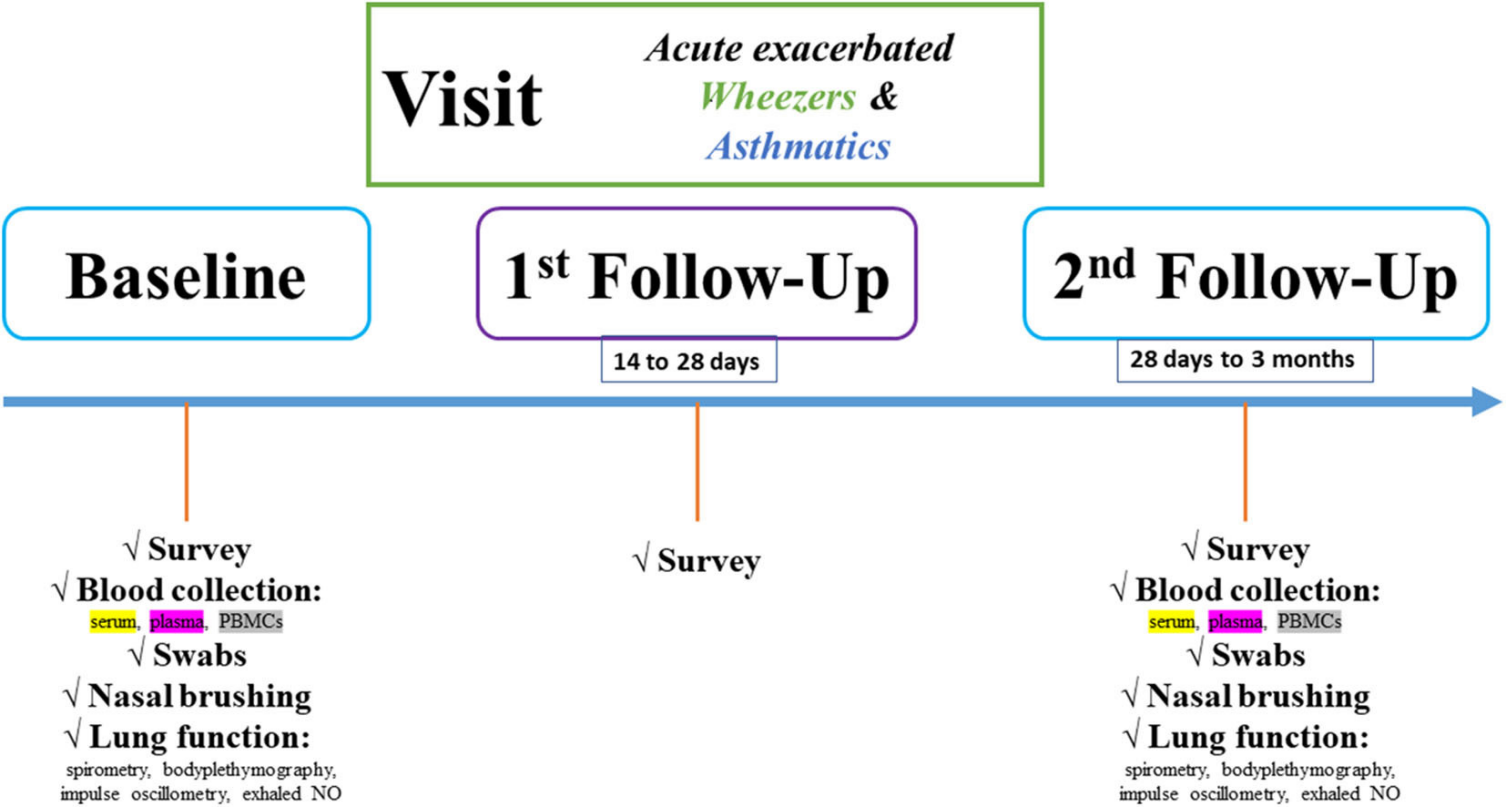
Flowchart of the pediatric exacerbation study network. Study design is characterized by baseline, first and second FU visits. At each study visit, different biomaterials and clinical item parameters will be obtained.

All individuals will be observed on the basis of two follow-up (FU) visits to recognize how the “active” and “non-active” phases are presented and differ, respectively (Figure 2).

In detail, at baseline and second FU visits, serum and plasma samples will be collected in monovettes purchased from Sarstedt®. Serum samples will be kept in the dark for 20 min before centrifugation. Plasma samples will be centrifuged upon collection (2.000×g for 15 min). Two-hundred microliter supernatant (serum/plasma) will be transferred to cryotubes, and the samples will be stored at −80°C until experimental use. Furthermore, the blood collection during both visits comprises also PBMC collection. Lithium-heparin/ethylenediaminetetraacetic acid blood tubes (Sarstedt, ORT) will be filled up with blood. The blood volume will be diluted with phosphate-buffered saline (Biochrom, ORT) and overloaded on Biocoll (Biochrom, ORT) in an appropriate ratio, as described in the literature. Afterward, centrifugation at 300 × g for 30 min will be taken place. Carefully, the PBMCs will be aspirated and transferred to a new column tube, and after several phosphate-buffered saline washing steps and cell calculating, the PBMCs will be diluted in fetal bovine serum, dimethyl sulfoxid (and DMEM) in an appropriate ratio as described in the literature and stored until experimental use. In addition, the nasal and pharyngeal swabs will be directly stored at −80°C until microbiome and fungiome analyses. The microbiome project is still under publication elsewhere. The methods will be shortly presented here. After DNA isolation of the swabs, a previously established protocol for 16S- and 18S-rRNA will be used. Region-specific primers will be chosen, and the region-of-interest will be transcribed and amplified through PCR steps. Finally, a barcode will be adapted to the amplified template and via NGS, the microbiome and fungiome libraries will be bioinformatically analyzed and correlated to the clinical item parameters. The microbiome project is still under publication elsewhere, and the fungiome project is currently in the establishing stage. The fungal sequencing steps will be adapted as published by Illumina® (https://emea.illumina.com/content/dam/illumina-marketing/documents/products/appnotes/its-metagenomics-app-note-1270-2018-001-web.pdf).

Moreover, through a brushing procedure, nasal epithelial cells will be isolated and used for functional analyses. For this, different cell culture models (2D submerged, organotypic 3D cell culture, and organoid models) will be established. These cell culture models will enable us to perform *in vitro* analyses of *in vivo* observed results derived from the entire study population, i.e., describing signaling pathways or evaluation of biomarkers. Moreover, induced sputum will be analyzed for cytokine and chemokine analyses. Furthermore, a questionnaire will be filled by subjects and custodians and a lung function test (including body plethysmography, spirometry, and exhaled NO for children beyond 5 years of age) will be performed at baseline and second FU visits. In addition, subjects (>2 years of age) will perform an impulse oscillometry. The questionnaire comprises different questions on the history of the child and the custodians, current clinics, including asthma scoring systems (Table 1). This questionnaire was based on a specific patient-related anamnesis questionnaire used in our clinics for many years when examining patients with respiratory symptoms. It was not necessary to validate the questions. The item parameters are sufficient enough to characterize the study population in different clinical phenotypes.

**Table 1:** Questionnaire used for the entire study population, not validated or published before, previously established, and currently still in use in the clinical routine.

**Questionnaire (summarized item parameters)**
i. Age and sex at study enrollment
ii. Place of birth, mode of birth, gestational weeks, birth height and weight
iii. Postnatal complications, including infections, mechanical ventilation
iv. Duration of breastfeeding
v. Vaccination
vi. Pet owner
vii. Allergic symptoms including itch, rhinitis, conjunctivitis, and duration of allergic symptoms
viii. Atopic eczema
ix. Family history for atopic disorders
x. Recurrent respiratory symptoms at preschool period
xi. Chronic medication and the response to this medication
xii. Asthma control test score
xiii. Current symptoms (e.g., cough, chest tightness, wheezing, and fever)

The study recruitment started in January 2018 and will be finished on 31/12/2021. All patients with typical symptoms requiring study enrollment will be presented at the emergency room of the (affiliated) university children's hospitals, and a study enrollment protocol will be obtained. Participating study centers are Center of Child and Adolescent Medicine, Helios University Hospital Wuppertal of Witten/Herdecke University, Germany and Children's Hospital, and Helios Hospital Niederberg, teaching hospital of University Hospital Essen/Germany. All participated subjects and/or their parents/legal guardians provided written informed consent. Healthy controls will be recruited mostly from children of teammates of the study centers, or the following alternatives may be considered for the recruitment of healthy children and adolescents, e.g., calls in social platforms, flyers for day-care centers, schools, private practices, social institutions, newspapers, and internet. These announcements will be separately reported to the ethics committees in advance.

A patient stratification in subgroups (atopic vs. non-atopic, transient vs. seasonal-dependent vs. multi-triggered vs. single-triggered vs. permanent wheezers, steroid-independent or steroid-dependent phenotypes) were based on clinical (questionnaire) and laboratory parameters (e.g., immunoglobulin E) (32).

This research received funding of the Faculty of Health at Witten/Herdecke University, Germany, and Helios Center for Research and Innovation by Helios Kliniken GmbH. The funding organizations had no role in study design or conduction, analysis, interpretation of the data, and decision to submit the manuscript for publication. Not any industry or private companies did not finance this study. It is an observational study of purely scientific interest without any interventional testing, including drug discovery or development. Randomization of the subjects or blinding of the study team are not necessary.

All analyzed biomaterials and data involving human participants are in accordance with ethical standards and with the Helsinki declaration (1964) and its later amendments or comparable ethical standards. Ethical approval and later amendment approvals are obtained by the Ethics Committees of Witten/Herdecke University and Ärztekammer Nordrhein, and the study is registered at the German Clinical Trials Register (DRKS). All experiments, including the data extraction and analyses, were and will be obtained pseudonymously. The obtained subject-relevant information and biomaterials were and will be separately coded with study-relevant numbers and codes. All subject-relevant data and experimental results will be filled in a database to perform correlation analyses. With this coding strategy, except the (deputy) study head, nobody may follow retrospectively to patient-sensitive information. All biomaterials were processed according to established internal standard operating procedures and were stored at −80°C or in liquid nitrogen until experimental use. The study recruitment is still ongoing, and the current subject and biomaterial numbers allow first pilot experiments and statistical analyses. The description of the collected data will be done on the basis of statistical parameters. Insofar as the distribution of the data enables, the comparison of the two groups will be carried out using analyses of variance and typical significance tests. Data will be presented as box plots with medians, interquartile ranges, minimum–maximum values or standard deviation, or standard error of the mean. In addition, the data will be presented and analyzed by heat maps, cluster analyses, point clouds, bar, and column charts. Moreover, sequencing results will be normalized within each group, and normalization methods, e.g., DeSeq2, will be performed as previously described by Anders and Huber (33). In addition, metagenomic and proteomic data will be analyzed by professional biostatisticians within our study network. The raw data will be uploaded on separate repository databases, as stated by the journal's requirements. For the experiments, age- and sex-matched stratification will be done between diseased and healthy groups in which each diseased patient will be compared with the same healthy children based on age and sex.

## Advantages and Pitfalls

This study is a prospective, multicenter, and observational cohort study conducted in two centers in Germany. A disadvantage of our study is the short observation period of our subjects, which is currently limited to 3 months. The study cohort can only be examined at two different time points after the baseline visit. The effects of exacerbation at the longitudinal level cannot be determined yet. Therefore, we are currently planning to establish a longitudinal study with the wheezers to be able to observe the mechanisms behind their development of bronchial asthma in the FU. One consideration here, for example, would be the recruitment of children when being infected with viral infections (e.g., influenza virus, respiratory syncytial virus, or coronavirus). Biomaterials could be collected at different points in time, for example, during exacerbation and FU. In addition, a further pitfall is the reduced motivation of parents and patients to attend FU visits, as is the case in many studies. This presents a major problem to achieve a significant subject number.

A further limitation is a regional dependency during the interpretation of the results. Our study cohort will be recruited in two urban centers, and a comparison cohort living in rural areas is currently not included. Here, supra-regional study centers should be included in the near future. Subsequently, there are also country-specific difficulties in the interpretation of the observed data. Another limitation is the lack of recruitment of adult asthmatics. Although we can compare the data with healthy children and adolescents, we do not have any reliable information on age dependency. We are currently planning an adult cohort and hope to submit the ethics proposal in the foreseeable future.

To handle the sampling procedure homogenously, we established standard operating procedure protocols where each sample should be processed, standardized upon biomaterial collection to reduce the pitfalls between persons. All study relevant materials, e.g., monovettes, swab materials, etc., were tested for suitability before recruiting. The nucleic acid concentration and the cell counts were appropriated during pilot sample processing.

In contrast, there is a large number of advantages to the study. Briefly, there is little data in the literature on infants with chronic bronchitis and adolescents with asthma exacerbation. Questionnaires can be used to identify risk factors that may have potentially led to exacerbation. In addition, impulse oscillometry ensures first lung function volumes in children beyond the age of 2 years, which we additionally collect in the course of the study. Phenotype and endotype comparisons between wheezers and asthmatics can be done, and potential diagnostic and therapeutic schemes can be outlined. The experiments are currently being carried out with the latest technologies and the experimental results. The current biomaterial number of approximately 2.500 samples ensures accurate statistical analyses. Biomarker research is currently one of the most important areas in translational research. Here, we can lay the first foundations with complex experiments and verify their clinical applicability. The large study network from a total of 12 centers offers valid feasibility of the experiments.

## Conclusion

Based on molecular and disease-specific pathways, a more sensitive and personalized diagnosis of asthma could be helpful. In fact, exacerbation cohorts, particularly in pediatrics, are difficult to establish. We were successfully able to establish a pediatric exacerbation study network in Germany, and the subject recruitment is still ongoing. We believe that the power of our study design and population and the performed experiments can be utilized to identify biomarkers that determine disease development at early stages, which allow stratifying patient's risk, the progression of the disease, and personalization of therapy regimens, i.e., sub-phenotyping or achieving insights into the underlying mechanisms that could become targets for novel therapies in the near future. All in all, our study population will open near doors for new research questions, which motivate us to better understand the exacerbation in detail. Due to the difficulty of recruiting exacerbated asthmatic children, a limitation is that we will need more international data to compare our findings and to describe regional-dependent phenotype changes. Our future aim is to establish an international pediatric exacerbation cohort from different centers worldwide to work on regional differences in the near future.

## Data Availability Statement

The datasets used during the current study were available from Malik Aydin on reasonable request at Center of Child and Adolescent Medicine, Helios University Hospital Wuppertal, Center for Clinical and Translational Research, Laboratory of Clinical Molecular Genetics and Epigenetics, Center for Biomedical Education and Research (ZBAF), Faculty of Health, Witten/Herdecke University.

## Ethics Statement

The studies involving human participants were reviewed and approved by Witten/Herdecke University (158/2017), Ärztekammer Nordrhein (NR 2019312) and Deutsches Register für Klinische Studien (DRKS00015738). Written informed consent to participate in this study was provided by the participants' legal guardian/next of kin.

## Author Contributions

MA and SW: concept, design, and had full access to all of the data in the study and take responsibility for the integrity of the data and the accuracy of the data analysis. MA, SL, AM-B, FK, AE, JP, and SW: acquisition, analysis, or interpretation of data. MA, FK, JP, and SW: drafting of the manuscript. MA, EN, SL, AM-B, WA, FK, AE, JP, and SW: critical revision of the manuscript for important intellectual content. AM-B, FK, AE, JP, and SW: supervision. All authors contributed to the article and approved the submitted version.

## Conflict of Interest

The authors declare that the research was conducted in the absence of any commercial or financial relationships that could be construed as a potential conflict of interest.
